# Extracorporeal cardio-pulmonary resuscitation in poisoning: A scoping review article

**DOI:** 10.1016/j.resplu.2023.100367

**Published:** 2023-02-18

**Authors:** Mingwei Ng, Zi Yang Wong, R. Ponampalam

**Affiliations:** SingHealth Toxicology Service, Singapore

**Keywords:** ECMO, ECLS, Extracorporeal CPR, Toxicology, Cardiac arrest, Poisoning, Overdose

## Abstract

**Background:**

Extracorporeal cardiopulmonary resuscitation (ECPR) represents last-line salvage therapy for poisoning-induced cardiac arrest but no review has focused on this specific area.

**Objective:**

This scoping review sought to evaluate the survival outcomes and characteristics of published cases of ECPR for toxicological arrest, with the aim of highlighting the potential and limitations of ECPR in toxicology.

Eligibility Criteria.

We searched PubMed and Cochrane for eligible papers from database inception to October 1, 2022 using the keywords “toxicology”, “ECLS” and “CPR”. References of included publications were searched to identify additional relevant articles. Qualitative synthesis was used to summarize the evidence.

**Results:**

85 articles were chosen: 15 case series, 58 individual cases and 12 other publications that were analyzed separately due to ambiguity. ECPR may improve survival outcomes in selected poisoned patients, although the extent of benefit is unclear. As ECPR for poisoning-induced arrest may have better prognosis compared to from other aetiologies, it is likely reasonable to apply ELSO ECPR consensus guideline recommendations to toxicological arrest.

Out-of-hospital cardiac arrest alone may not be sufficient grounds to deny ECPR if effective resuscitation had been promptly instituted. Poisonings involving membrane-stabilizing agents and cardio-depressive drugs, and cardiac arrests with shockable rhythms appear to have better outcomes. ECPR may permit excellent neurologically-intact recovery despite prolonged low-flow time of up to four hours. Early ECLS activation and pre-emptive catheter placement can significantly shorten time-to-ECPR and possibly improve survival.

**Conclusion:**

As effects of poisoning may be reversible, ECPR can potentially support poisoned patients through the critical peri-arrest state.

## Introduction

Extracorporeal life support (ECLS) arguably represents the last-line rescue therapy for severe near-fatal cardiogenic shock arising from drug intoxication when medical therapy fails^1^. As the clinical effects of poisoning are usually temporary and reversible, ECLS can be used as a salvage therapy to maintain perfusion to support the vital organs, permit endogenous drug metabolism and elimination and allow time for administration of antidotes if available^2^.

## Rationale

While cardiac arrest represents the most extreme case of cardiogenic shock, much less is known about the utility of ECLS for cardiac arrest from drug intoxication during active ongoing cardiopulmonary resuscitation (CPR) – otherwise known as extracorporeal CPR (ECPR). The Extracorporeal Life Support Organization (ELSO) defines ECPR as the application of rapidly-deployed veno-arterial extracorporeal membrane oxygenation (ECMO) in patients in whom conventional CPR is unsuccessful in achieving sustained return of spontaneous circulation (ROSC)^3^. As Voicu *et al* (2022) pointed out in a most recent review article, the indications, timing and outcomes for ECLS in cardiotoxic poisoning – least to say the role of ECPR in poisoning-induced cardiac arrest – remain speculative due to the limited data, heterogeneity and multi-drug nature of most poisonings, and the fact that conducting randomized human trials is virtually impossible^4^.

Thus far, no systematic review has focused on the role of ECPR specific to drug intoxication and evidence has been purely observational to date^5^. Herein lies the clinical dilemma: without a clear understanding of its optimal timing and indications, ECPR might be initiated either too early when ROSC might still have been possible with conventional CPR, exposing the patient unnecessarily to immense risks from such an invasive procedure; or too late, after the onset of irreversible end-organ and brain damage^6^. As such, this scoping review seeks to help inform clinicians on the limitations and potential of ECPR for poisoning-induced cardiac arrest.

## Objective

The objective of this scoping review was to evaluate the survival outcomes and characteristics of published cases of ECPR for poisoning-induced cardiac arrest. Primary outcome of interest was survival. Secondary outcomes were functional outcomes and duration of ECLS support required. Complications from treatment were also documented where reported.

## Methods

### Eligibility criteria

#### Study types

All sources including observational studies, reviews, letters, comments, case reports, meeting abstracts and conference proceedings in any language were included without date restriction up to October 1, 2022 when the most recent search was executed. The search was not limited by publication status or by type as relevant literature was already scarce. The search was also not limited to English language to avoid language bias, particularly because several institutions hailing from non-English native-speaking countries like France have amassed considerable experience with ECPR and published prominent landmark articles.

#### Participants

Studies were eligible if they involved human cases of poisoning leading to refractory normothermic cardiac arrest. Articles that reported paediatric cases were included if relevant. Studies based on experimental animal models were excluded.

Cases of cardiogenic shock without cardiac arrest were excluded. Cases of cardiac arrest confounded by severe hypothermia were also excluded, as the role of ECPR for rewarming in hypothermic cardiac arrests has been firmly established and prognoses and management considerations for accidental hypothermic cardiac arrest differ uniquely.

Given the sometimes fleeting and transitory nature of cardiac arrest, cases where there was ambiguity over the temporal sequence of events (whether the patient had regained ROSC with conventional CPR and was then started on ECLS, or remained pulseless prior to ECLS) were retrieved but analysed separately. This included cases that reported transient ROSC with multiple refractory cardiac arrests, and cases where ECLS cannulation was performed in peri-arrest patients with conventional closed-chest CPR ongoing for agonal low-flow cardiac output.

#### Interventions

Studies that discuss the role of extracorporeal cardiopulmonary resuscitation were eligible. Based on the ELSO definition of ECPR, cases that focused on the use of veno-venous ECMO (indication being respiratory failure rather than circulatory collapse) and cases of cardiac arrest that were successfully resuscitated with conventional CPR and had sustained ROSC prior to ECLS were excluded.

#### Outcome measures

Studies needed to document at least one of the primary or secondary outcomes to be eligible. Primary outcome of interest was survival. Secondary outcomes were functional outcomes and duration of ECLS support required. Complications from treatment were also documented where reported.

## Search strategy

The authors followed the Preferred Reporting Items for Systematic reviews and Meta-analyses extension for Scoping Reviews (PRISMA-ScR) guideline. This scoping review was not eligible for inclusion in PROSPERO as it constituted a scoping review.

**Fig. 1:**
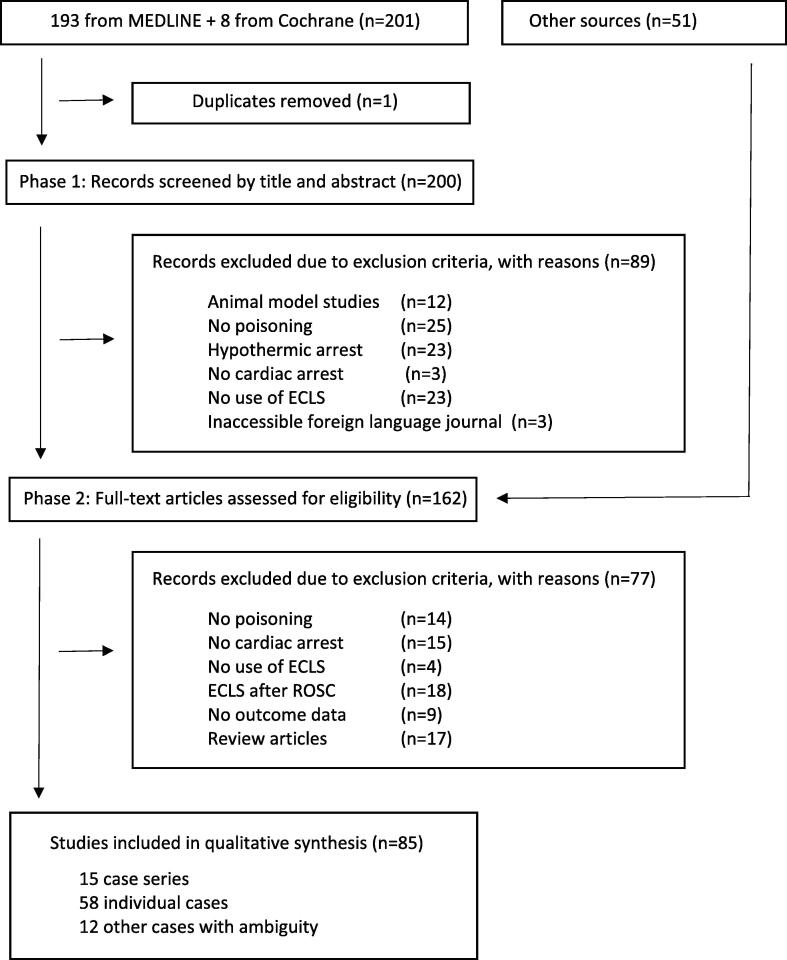
**Search Strategy and Flow Diagram. Search Strategy.** (toxicol* OR poisoning OR overdose OR intoxica*) AND (extracorporeal OR ECLS OR ECPR OR ECMO OR cardiopulmonary bypass) AND (cardiopulmonary resuscitation OR CPR OR cardiac arrest OR ECPR).

The authors accessed PubMed and Cochrane Library to identify eligible papers from database inception to October 1, 2022. A search strategy was developed in conjunction with content experts and librarians, using the following keywords: (toxicol* OR poisoning OR overdose OR intoxica*) AND (extracorporeal OR ECLS OR ECPR OR ECMO OR cardiopulmonary bypass) AND (cardiopulmonary resuscitation OR CPR OR cardiac arrest OR ECPR) [Fig. 1]. Other sources like Google Scholar were also searched. In addition, a secondary search of the citations and references of all included articles was performed to identify additional germane articles that were not indexed by PubMed or Cochrane Library and which would therefore have been missed by the primary search strategy. The protocol for this scoping review was not registered or published in advance.

Two authors (MN, ZW) independently carried out screening for eligibility, blinded to authors and journal names. Differences were resolved by discussion and consensus, or when necessary, a senior third reviewer (RP). All retrieved citations were exported and duplications were removed. Studies were filtered according to inclusion criteria and the authors reviewed the titles and abstracts of all studies before retrieving and reviewing the full texts. A data abstraction form was used to standardize data retrieval after a pilot version was trialled among the data abstractors on three cases. Study characteristics (such as author, country, year of publication, study design and study period), subject characteristics (number, inclusion criteria, age, gender, type of offending agent, dose, route, co-ingestions, presence of shockable rhythm upon arrest), treatment characteristics (treatments instituted, requirement for transfer, time to treatment) and outcome characteristics (survival outcomes, functional outcomes) were independently abstracted by the two data abstractors (MN, ZW) for each included study. No significant abstraction differences were found between authors. An independent author (MN) then merged the data into a single database to ensure uniformity. Efforts were made to reach out to the corresponding authors of selected articles where there was ambiguity to make important clarifications. Qualitative synthesis was used to summarize the evidence.

In this scoping review, no risk of bias assessment or other assessment of methodological quality was performed on the identified studies.

## Results

The primary search strategy yielded 201 eligible articles. A further 51 articles were identified to be of relevance based on the references and bibliography of these chosen articles [Fig. 1]. Three foreign-language journals could not be accessed and their corresponding full-text articles were thus not included. 162 articles were selected for full-text review in Phase Two.

17 of the articles excluded in Phase Two were review articles – some of which discussed ECLS for poisoning-induced cardiotoxicity in general while others focused on specific poisons. These articles mostly recommended early initiation of aggressive measures like ECLS for poisoned patients with severe cardiotoxicity once standard resuscitative measures have failed^7^, although Baud cautioned that it is difficult to draw any conclusions regarding the efficiency or indications of ECLS from the scarce literature^8^.

### Study characteristics

Of the 85 studies eventually included in the qualitative synthesis, 15 articles were case series [Table 1][9], [10], [11], [12], [13], [14], [15], [16], [17], [18], [19], [20], [21], [22], [23] while 58 were individual cases [Table 2**,** Supplement 1][24], [25], [26], [27], [28], [29], [30], [31], [32], [33], [34], [35], [36], [37], [38], [39], [40], [41], [42], [43], [44], [45], [46], [47], [48], [49], [50], [51], [52], [53], [54], [55], [56], [57], [58], [59], [60], [61], [62], [63], [64], [65], [66], [67], [68], [69], [70], [71], [72], [73], [74], [75], [76], [77], [78], [79], [80], [81]. Most of the studies (49/73, 67.1 %) were published in the last ten years, with the oldest published case dating back to 1984. 12 other publications were analysed separately due to ambiguity over the temporal sequence of events (whether ECPR was necessary to achieve ROSC or whether ROSC had been achieved with conventional CPR instead) [Supplement 2][82], [83], [84], [85], [86], [87], [88], [89], [90], [91], [92], [93].

**Table 1:** Case Series of ECPR for Poisoning-Induced Cardiac Arrest.

#	Year	Author, country Hospital of interest	Study Type Language	Sample	Study period	Size	Key findings
9	2000	Massetti, FRA Caen University Hospital	Letter to the Editor English	Cardiopulmonary bypass for severe drug intoxication	N.A.	7	5/7 survival • 1 survivor required fasciotomy for lower leg compartment syndrome
10	2001	Babatasi, FRA Caen University Hospital	Cohort French	ECLS for cardiac arrest following ingestion of cardiotoxic drugs	May 1997 – Mar 2000	6	4/6 survival • First 2 patients died of multiorgan failure following delays in time to ECPR • All survivors had no sequelae
11	2005	Massetti, FRA Caen University Hospital	Cohort English	ECLS for refractory cardiac arrest	Jun 1997 – Jan 2003	6	4/6 survival in “medical intoxication” group • Higher survival compared to non-poisoning arrests (4/34; 11.8%)
12	2007	Megarbane, FRA Lariboisiere Hospital	Cohort English	ECLS for refractory cardiac arrest	Jul 2003 – Jul 2005	12 • 8 OHCA • 4 IHCA	3/12 survival • All 3 survivors had neurologically-intact recovery o Flecainide/acebutolol: CPR duration 30min, left ICU Day 12 – despite markedly elevated plasma lactate of 39.0mmol/L before cannulation! o Acebutolol: CPR duration 100min, left ICU Day 13 o Acebutolol sustained-release: CPR duration 180min, left ICU Day 14 • CPR duration before ECLS initiation was long (120min, 45-180min) because cardiothoracic surgery was based at neighbouring hospital • Toxins: acebutolol (*n*=3), flecainide (*n*=2), chloroquine (*n*=2), verapamil (*n*=2), propranolol (*n*=1), dextropropoxyhen (*n*=1), colchicine (*n*=1) • Successful cannulation in all patients but failure to achieve targeted extracorporeal flow in 2/12 • Mean ECLS duration was 56 hours (5-108 hours) • Higher survival compared to non-poisoning arrests (0/5; 0.0%)
13	2007	Sodeck, AUT Vienna General Hospital	Case series English	Unstable bradycardia	Mar 1994 – Mar 2004	2	2/2 survival among intoxicated patients “stabilized only by cardiopulmonary bypass”
14	2009	Daubin, FRA Caen University Hospital	Cohort English	Drug-induced prolonged cardiac arrest/refractory shock	1997 – 2007	7	5/7 survival • All 5 survivors had neurologically-intact recovery (CPC 1)
15	2011	Masson, FRA Caen University Hospital	Comparative Cohort English	Persistent cardiac arrest /severe shock following drug intoxication	Jan 1999 – Jun 2010	3	3/3 survival
16	2015	Brunet, FRA Caen University Hospital	Cohort English	ECLS for refractory cardiac arrest/shock	Apr 2003 – Apr 2013	6 • 2 OHCA • 4 IHCA	4/6 survival • IHCA (3/4 survival) had more favourable outcomes to OHCA (1/2 survival) • Higher survival compared to arrests from acute coronary syndrome (2/12; 16.7%) or other causes e.g., drowning, pulmonary embolism (0/10; 0.0%)
17	2015	Rousse, FRA Lille University Hospital	Cohort English	ECLS in refractory OHCA	Dec 2009 – Dec 2013	9 • 9 OHCA	0/9 survival • Median age 46.9years • All were witnessed arrests, no-flow duration <5min • 4/9 had moderate-to-severe hypothermia (core T◦c <32◦c) but demised • Toxins: beta-blockers (*n*=2), calcium antagonists (*n*=1), SSRIs (*n*=2), benzodiazepines (*n*=2), poly-intoxication (*n*=2)
18	2015	Wang, USA ACMT ToxIC Registry	Cohort English	ECLS cases reported to ACMT ToxIC registry	Jan 2010 – Dec 2013	4	4/4 survival (See **Table 1.1**) • *Unclear if patients required ECPR to achieve sustained ROSC, or if ROSC was achieved with conventional CPR prior to ECMO initiation
19	2016	Baud, FRA Hospitals in Assistance Publique-Hopitaux de Paris (APHP) group	Cohort Spanish	ECLS for drug-induced refractory cardiogenic shock and cardiac arrest	2002-2012	71 • 45 OHCA • 26 IHCA	8/71 survival • 5/45 (11.1%) for OHCA; 3/26 (11.5%) for IHCA • Dose ingested was “very high” in all cases • Higher survival in cardiotoxic (10%) vs non-cardiotoxic drug-induced arrests o Survival lowest for chloroquine, colchicine, verapamil o Survival poor for sedative/ hypnotic subgroup, suggesting arrest occurred because of anoxia o Survival highest for beta-blockers, antiarrhythmics
20	2017	Pozzi, FRA Louis Pradel Cardiogenic Hospital	Cohort English	ECLS for refractory cardiogenic shock/IHCA due to cardiotoxic poisoning	Jan 2010 – Dec 2015	3 • 3 IHCA	1/3 survival • Only survivor had neurologically-intact recovery (CPC 1) • All intoxications were with beta-blockers, calcium channel blockers or membrane-stabilizing agents • All arrests were witnessed (no-flow time = 0 min) • Mean low-flow time was 67.5 min
21	2019	Lewis, USA California Poison Control System	Cohort English	ECLS cases reported to California Poison Control System	1997 – 2016	3	2/3 survival (See **Table 1.1**) • *Unclear if patients required ECPR to achieve sustained ROSC, or if ROSC was achieved with conventional CPR prior to ECMO initiation
22	2021	Pozzi, FRA Louis Pradel Cardiogenic Hospital	Cohort English	VA-ECMO for drug intoxication-induced refractory cardiogenic shock/cardiac arrest	Jan 2007 – Dec 2020	7 • 1 OHCA • 6 IHCA	3/7 survival • Significantly lower survival compared to 88.0% survival for ECLS for poisoning-induced cardiogenic shock
23	2022	Duburcq, FRA Lille University Hospital	Cohort English	VA-ECMO for drug intoxication	Jan 2014 – Dec 2020	12 • 5 OHCA • 7 IHCA	3/12 survival • All were witnessed arrests and received immediate CPR • IHCA (2/7 survival) had more favourable outcomes to OHCA (1/5 survival) • Initial rhythm was asystole in 11/12 • 8 demised within 48h while on ECLS (multiorgan failure prompting withdrawal of ECLS, *n*=5; brain death, *n*=3); the last died after weaning off ECMO due to severe hypoxic ischemic encephalopathy • Low-flow duration shorter in survivors (45min (40-60)) than non-survivors (77.5min (65-100)) (p=0.02)

**Table 2:** Summary of Individual Cases of ECPR for Poisoning-Induced Cardiac Arrest.

	All [median (%)]; *n* = 58 (100.0%)	Survived; n=48 (82.8%)	Demised; n=10 (17.2%)
Age (years)	25.0 (17.8-43) - Missing data in 2 cases	26.0 (18.5-45.3) - Missing data in 2 cases	25.0 (6.5-39.3)
Sex (male)	24 (41.4%)	19 (39.6%)	5 (50.0%)
Single-agent intoxication	45 (77.6%)	38 (79.2%)	7 (70.0%)
Poisoning by cardiotoxic drug or membrane-stabilizing agent (MSA)	41 (70.7%)	34 (70.8%)	7 (70.0%)
• Cardiotoxic drug/MSA		Verapamil (3), diltiazem, amlodipine (2) Sotalol, propranolol (2), carvedilol, metoprolol Lignocaine (2), bupivacaine (2) Yew (7), aconite Flecainide (2), ajmaline (2), cibenzoline (3), propafenone Diphenhydramine (2) Amitriptyline (4), nortriptyline	Verapamil Metoprolol, betaxolol BupivacaineYew Digoxin Flecainide, propafenone
• Non-cardiotoxic drug/MSA		Aluminium phosphide (2) Aspirin/risperidone/flunitrazepam Caffeine (2) Ethylene glycol MDMA/cannabis White gilled mushrooms (2) Chinese herbal medications Venlafaxine Boric acid/mirtazapine/sennosides Chloroquine Brown recluse spider	Aluminium phosphide Aspirin Cyclophosphamide Loperamide
In-hospital cardiac arrest	55 (96.5%) - Missing data in 1 case	45 (95.7%) - Missing data in 1 case	10 (100.0%)
Low-flow time (minutes) • Time from arrest to ECLS flow	60.0 (36.5-90.0) - Missing data in 19 cases	63.0 (39.8-90.0) - Missing data in 14 cases	49.0 (35.0-89.0) - Missing data in 5 cases
Duration of ECLS support (hours)	70.0 (46.5-96.0) - Missing data in 4 cases	70.5 (48.0-96.0) - Missing data in 2 cases	36.0 (4.0-102.0) - Missing data in 2 cases

### Case series

15 case series involving 158 patients were identified. Among the case series, two were from the United States^18,^^21^, one from Austria^13^ and the remaining 12 were from France^9-12,^[19], [20], [22], [15], [16], [17]. Of the French articles, six were from Caen University Hospital^9-11,^[14], [15], [16] while Lille University Hospital^17^^.^^23^ and Louis Pradel Cardiogenic Hospital^20,^^22^ contributed two each. These were all urban-based tertiary institutions.

All selected case studies were retrospective observational studies. None of the studies matched cases with controls, although one retrospective observational cohort study by Masson *et al* drew comparisons between consecutive poisoned patients admitted to two university hospitals – one highly-practiced in ECPR, while the other had no access. Masson concluded that ECPR for severe poisoning-induced cardiovascular shock or collapse was associated with lower mortality compared to conventional treatment^15^. Three cases series^11,^[12], [16] evaluated ECPR outcomes for poisoning-induced cardiac arrest against ECPR for non-toxicological arrest – all three found more favourable survival outcomes for the former.

All 15 case series identified evaluated survival as their primary outcome. ECPR survival rates cited ranged from 100 %[13], [15], [18] to 0 % in Rousse *et al*^17^, an out-of-hospital cardiac arrest (OHCA) cohort in which all nine patients demised despite all nine being witnessed arrests with no-flow time less than five minutes and despite four patients having had moderate-to-severe hypothermia which ought to have been protective^17^. The largest of the case series was a multi-centre study by Baud *et al* (*n* = 71) which found a survival rate of 11 %^19^.

Three case series[16], [19], [23] drew comparisons between out-of-hospital (OHCA) versus in-hospital cardiac arrest (IHCA) but their findings conflicted. While Baud *et al* found similar survival-to-discharge rates for IHCA (3/26; 11.5 %) and OHCA (5/45; 11.1 %)^19^, Brunet^16^ and Duburcq^23^ observed more favourable outcomes for IHCA.

### Individual cases

Virtually all of the individual cases (55/57, 96.5 %) cases were witnessed IHCAs, although two cases first had OHCA and achieved ROSC with conventional CPR before suffering another IHCA requiring ECPR^51,^^73^. The two remaining OHCA case reports were both witnessed and received immediate CPR from trained paramedics – both survived with intact neurological function despite being OHCAs[71], [79].

Among the individual cases, 41/58 (70.7 %) involved poisoning by cardiotoxic drugs or membrane-stabilizing agents. They were mostly single-agent intoxications (45/58, 77.6 %). Low-flow duration (time from cardiac arrest to ECPR) ranged from three minutes^77^ to four hours^32^, with a median of 60 minutes [Table 2]. Five cases (5/41, 12.2 %) had low-flow durations exceeding 120 minutes but all five had excellent functional recovery. 48/58 (82.8 %) of the individual cases described successful resuscitation with ECPR [Table 2], all of which had complete neurological recovery and no functional impairment upon discharge except for six cases with residual deficits^29,^[41], [43], [54], [58], [65].

## Discussion

There have been many advances in ECLS over the past 30 years, increasing the opportunity to use this approach to salvage patients with poisoning-induced cardiac arrests. Up to 1980 s, cardiopulmonary bypass support necessitated median sternotomy to permit right atrial venous cannulation^26^, vastly different from current extracorporeal techniques which are far less invasive and do not require interruption of external cardiac massage. Napp *et al* described a novel approach to the management of life-threatening venlafaxine overdose which involved escalating veno-arterial ECMO to veno-arterial-pulmonary arterial ECMO by introducing a third cannula into the pulmonary artery – this first-in-man, fully-percutaneous complete heart–lung bypass set-up^57^ is particularly fascinating because it demonstrates how clinical toxicology has become the medium in which advancements in intensive care develop.

However, despite increasing adoption as a last-ditch salvage modality for cardiac arrest in recent years, the evidence for ECPR in the context of poisoning-induced arrests remains limited to predominantly case reports and a few small observational cohort studies. Although reported ECPR survival rates vary widely, a significant number of studies have described instances of functional and neurologically-intact recovery with ECPR. This suggests that optimal patient selection is key and ECPR still shows much promise in the correct carefully selected patient subgroup^94^.

For instance, ECPR for cardiac arrest from cardiotoxin or membrane-stabilizing agent poisoning in particular appears to have better prognosis^19^, as the evidence is strongest in this group. Patients presenting with cardiac arrest from shockable rhythms (ventricular fibrillation or pulseless ventricular tachycardia) also appear to have better outcomes (30/33, 90.9 %) compared to non-shockable rhythms (18/25, 72.0 %). This is consistent with cardiothoracic literature which found that shockable rhythms suggest retained myocardial viability and are therefore associated with improved ECPR outcomes^96^.

Three case series separately found that ECPR for poisoning-induced cardiac arrest has better prognoses compared to ECPR for non-toxicological cardiac arrest. The potentially reversible nature of most poisonings means that ECPR support can be used to maintain perfusion and provide circulatory support until the offending drugs are metabolized or eliminated and their effects reversed^2^ (“bridge-to-recovery”). In addition, patients that present with cardiac arrest from poisonings tend to be younger with no pre-existing co-morbidities. They are therefore more likely to have a better prognosis compared to older patients suffering cardiac arrest from organic causes^95^. This would also imply that it is reasonable to apply general ECPR recommendations put forth by ELSO consensus guidelines (such as consider commencing cannulation after 10–20 minutes of failed resuscitation efforts, or aim to establish ECLS flow within 60 minutes of cardiac arrest onset^6^) in the context of toxicological arrests as well. One could possibly even be more optimistic and aggressive in such poisoning cases, given that one can expect better prognoses than non-toxicological arrest.

### OHCA vs IHCA

Fewer, shorter delays to specialized care are expected in a resource-rich inpatient setting for IHCAs. This anticipation that survival outcomes from IHCA should outdo OHCA has likely led to institutional practices which bias IHCA over OHCA when assessing candidacy for ECPR. It is therefore surprising that Baud *et al* found no differences in survival outcomes between OHCA and IHCA^19^, and that both OHCA cases among the individual cases survived with full neurological recovery^71,^^79^. This suggests that perhaps ECPR for poisoning-induced arrest should not be denied solely on the basis of OHCA versus IHCA, but should instead depend on whether the cardiac arrest was witnessed and whether prompt and effective resuscitation had been instituted. Ultimately, even ECPR cannot reverse organ failure that has already developed. Outcomes are therefore still very much predicated on the patient receiving immediate and effective good-quality resuscitation to prevent irreversible organ failure from occurring prior to initiation of ECPR.

### Low-Flow time

Duburcq *et al* highlighted that low-flow time (time from cardiac arrest to ECPR) was shorter in survivors compared to non-survivors and recommended that shortening the timing to ECPR initiation ought to be made a key priority^23^. Although ELSO ECPR recommendations state that arrest-to-ECLS flow time should not exceed an hour^6^, Rygnestad *et al* and Baum *et al* in particular reported survival with intact neurological status despite low-flow durations of 240 minutes^32^ and 225 minutes^44^ respectively. Of course, individual case reports are likely to be plagued by publication bias but the findings nonetheless do lend credence to the argument that clinicians should continue resuscitation efforts and avoid calling the code too early in cases of witnessed toxicological arrest that received immediate CPR. In addition, it appears that protracted CPR and prolonged low-flow duration in toxicological arrests should not deter initiation of ECPR either.

On the other end of the spectrum, Yasuda *et al* described a case of life-threatening caffeine overdose in which low-flow time was only three minutes^77^. This feat was attributed to the early recognition of the life-threatening nature of the poisoning by the team and pre-emptive placement of catheter sheaths for ECLS that facilitated immediate introduction of ECPR support upon arrest^77^. Labarinas *et al* likewise reported nine minutes – in this case, the ECLS team had likewise been consulted early due to refractory hypotension and was already at the bedside when the patient developed sudden ventricular fibrillation arrest^60^. Indeed, survival odds deteriorate drastically once the poisoned patient has slipped into cardiac arrest – Pozzi *et al* reported markedly poorer survival for ECLS for poisoning-induced cardiac arrest (42.9 %) compared to poisoning-induced cardiogenic shock (88.0 %)^22^. As such, it is imperative that upon recognition of impending hemodynamic collapse, clinicians activate the ECLS team as early as possible to offer patients the best chance of survival. This recommendation is echoed by the ECPR Guideline Consensus Statement from ELSO which emphasized that early assessment of ECPR candidacy is crucial and it is in fact reasonable to start ECLS first as a “bridge-to-seeking further information”^6^ – especially in poisoning cases where incident circumstances are often unclear and information can be unreliable.

### Offending toxins

Cardiopulmonary bypass support for poisoning-induced cardiac impairment was first used predominantly for overdoses from cardio-depressive drugs (like beta-blockers, calcium channel antagonists) and membrane-stabilizing agents (Class I antiarrhythmic agents such as flecainide and bupivacaine)^97^. Current evidence for ECLS remains strongest for these drug classes. That this could be extrapolated to poisoning-induced cardiac arrest as well: survival was strongly related to class of drug ingested and highest after overdosing on cardiotoxic drugs like beta-blockers and anti-arrhythmics^19^. Baud *et al* further noted that outcomes for cardiac arrest from sedative-hypnotic overdose were especially dismal^19^. It was hypothesized that this is likely because ECLS is most effective as a form of mechanical circulatory support for cardiotoxic cardiac failure, whereas arrest in sedative-hypnotic overdoses had likely occurred due to global anoxia or cerebral hypoxia rather than cardiotoxicity^19^.

### ECLS capability

Patients who first presented to a peripheral hospital, arrested and required secondary transfer to an ECLS-capable centre with chest compressions ongoing had outcomes (5/6 survival, 83.3 %) nearly identical to cases that did not require secondary transfer (42/51 survival, 82.4 %) [Table 2]. Centres with no ECLS capability should therefore consider expedient transfer to an ECMO-capable centre in the vicinity while the patient is maintained on mechanical CPR for ECLS, provided that irreversible organ failure is unlikely to have occurred. Notably, Marano *et al* described an “on-site” ECPR concept where the patient is promptly cannulated, commenced on ECLS in the emergency room of a peripheral hospital with support from the ECLS team and transferred to the ECLS-capable centre once stabilized^62^. However, successful implementation of such a strategy would not only demand training multi-disciplinary teams of clinicians, perfusionists and support staff but also require significant coordination, logistical planning, equipment acquisition and careful preparation across multiple centres^7^.

It is noteworthy that criticism has been levelled on Masson *et al*^15^ for having significant overlap with Daubin *et al*^14^. Both studies drew from the same patient database of the same centre (Caen University Hospital) and had major overlaps in their study periods of interest as well^7^. As early as in 2001, Babatasi *et al* had published a small case series (*n* = 6) of ECLS for poisoning-induced arrests at Caen between 1997 and 2000^10^. It is unclear if this case series included the same patients that Massetti described in his “Letter to the Editor” in 2000^9^. In 2005, Massetti *et al* similarly highlighted his experience at Caen between 1997 and 2003 with cases that required ECLS for prolonged cardiac arrest^11^. More recently, Brunet *et al* re-explored the use of ECLS in Caen University Hospital again from 2003 to 2013^16^.

This disproportionate over-representation of survival cases from a select few institutions like Caen would amplify bias for ECPR. Although the study findings only reflect the results of ECPR in experienced hands at a single high-volume institution and may thus not accurately parallel the outcomes achievable by other less-practiced institutions^7^, the results do nonetheless affirm the tantalizing potential that ECLS has to offer the field of toxicology.

### Bridge-to-Transplant

Although this review paper focussed on survival and functional outcomes as the primary and secondary outcomes of interest, other outcomes are worth exploring. While complete myocardial recovery is often hoped for, the patient may sometimes still have severe residual cardiac function impairment necessitating further mechanical support from ventricular assist device or even orthotopic heart transplant^90^. In such extraordinary cases, ECLS should be viewed as “bridge-to-VAD/transplant” rather than as having failed.

Vivien *et al* also discussed an unfortunate patient who had flecainide and betaxolol poisoning and succumbed to brain death despite early ECLS. However, ECLS helped maintain vital organ perfusion and permitted successful recovery of the patient’s heart, liver and kidneys for allograft transplant^34^. Given that patients who suffer from poisoning-induced cardiac arrest are generally younger, healthy and have few co-morbidities, the immense potential for organ donation in these group further strengthens the case for aggressive resuscitation with ECLS.

### Resource utilization

While ECPR is resource-intensive and prohibitively expensive, St-Onge *et al* used various modelling tools to evaluate its cost effectiveness. Assuming 27 % survival in poisoned cardiac arrest patients treated with ECPR (an estimate derived from non-toxicological cohorts), St-Onge estimated that cost per life year ($34,311/life-year) was less than the $50,000-$100,000 per quality-adjusted life-year gained that was often cited in medical literature^98^.

Weiner *et al* carefully reviewed the cases from the ELSO registry and noted that prolonged ECMO runs were unlikely to be required specifically for poisoning-induced cardiac arrest^99^. Although Weiner had evaluated both poisoning-induced refractory cardiogenic shock and cardiac arrest and only 34/104 cases (32.7 %) had pre-ECMO cardiac arrest, the median duration of ECLS support in this cohort of 68 hours closely mirrored the 70-hour median duration of ECLS support among poisoned individual cases as reported in this review^99^.

## Strengths and limitations

This study represents the first and most current summary of the varied published evidence relating to ECPR for poisoning-induced cardiac arrest specifically. The strengths of this review are the systematic search technique, comprehensive data extraction and in-depth qualitative synthesis. However, this study has several limitations as expected of a retrospective literature review. Case reports and the occasional case series from various authors hailing from different centres of diverse clinical settings have together made up an extremely heterogenous cohort, while the lack of a standardized manner for reporting of events has also limited the ability to analyse findings.

For instance, lack of standardized definitions meant that some authors describe ECMO as a “veno-venous method of providing oxygenation” that was distinct from ECLS^8,^[95], [100] while others used the terms ECLS and ECMO interchangeably^5,^^22^. Indeed, Baud highlighted that various extracorporeal techniques have been developed but the same terms are often used with different meanings^8^. These subtle variances in terminology not only make defining the keyword search terms challenging, but also introduce much ambiguity when interpreting whether ECLS was truly instituted for circulatory collapse or for other indications such as respiratory failure.

Most of the germane articles in existing literature thus far are descriptive case reports and case series without comparisons drawn between ECLS and conventional treatment. This limits the utility of this scoping review to an observational nature. Moreover, the overwhelming majority of published cases reported successful positive outcomes. This is almost certainly due to the combined effects of deliberate and careful patient selection, publication bias and over-representation of successful cases, rather than proof that ECPR is truly a “Hail Mary” panacea capable of miraculously salvaging all patients. To this end, international multi-centre collaborative registries that track both ECPR successes and failures alike are less susceptible to the effects of publication bias and would represent potential areas for future collaboration and research.

## Conclusion

As poisonings represent a potentially reversible cause of arrest, ECPR may therefore be used to support the patient through the critical peri-arrest period. By maintaining vital organ perfusion, ECPR permits more time for drug clearance to enhance the patient’s chances of survival. Cardiac arrest from poisoning with cardiotoxic agents like beta-antagonists and membrane-stabilizing agents have more published evidence for successful ECPR outcomes. While ECPR is best instituted early as shorter low-flow times yield better outcomes, neurologically-intact and functionally-intact survival have been achieved even after prolonged normothermic ECPR runs of up to four hours^32^. ECPR shows great promise as potential salvage therapy for refractory cardiac arrest from poisoning, albeit with the caveat that it has yet to be supported by strong robust evidence.

## Funding

No funding or sponsorships were received for this study. All of the authors declare that they do not have any competing interests.

## Appendix

See Fig. 1 and Table 1, Table 2.
