# Supplementary material for: Extracorporeal cardio-pulmonary resuscitation in poisoning: A scoping review article
Source: Resusc Plus. 2023 Feb 18;13:100367. doi: 10.1016/j.resplu.2023.100367 (PMC9969255; doi:10.1016/j.resplu.2023.100367)
Supplement: Supplementary data 1 [file mmc1.docx]

Supplementary 1: Individual Cases of ECPR for Poisoning-Induced Cardiac Arrest

| # | Year | Author  Country | Study type  Language | Age, sex | Toxins (dose) | IHCA vs OHCA | Time from arrest to ECPR | Transfer to ECLS facility required | Intubation duration | Survival outcomes | Remarks   - Peak levels |
| --- | --- | --- | --- | --- | --- | --- | --- | --- | --- | --- | --- |
|  |  |  |  |  |  | VT/VF | ECLS duration |  | ICU LOS | Complications |  |
| 24 | 1984 | Noble  GBR | Case report  English | 58M | Lignocaine (2g) – intraoperative during CABG grafting | IHCA | Immediate | No | 2d | Discharged @ D14 completely well | ECLS immediately restarted after accidental lignocaine administration into bypass circuit during CABG grafting |
|  |  |  |  |  |  | No | 45m |  | N.A. | Nil |  |
| 25 | 1989 | Hendren  GEO | Case report  English | 2M | Verapamil SR (1.4g) | IHCA | 1h 29m | No | N.A. | Demised @ D1 |  |
|  |  |  |  |  |  | No | 3h 45m |  | 15h 30m | Cerebral oedema, visceral congestion |  |
| 26 | 1989 | Long  USA | Case report  English | 27F | Bupivacaine (75mg) and lidocaine/ epinephrine (525mg) for nerve block | IHCA | 1h 15m | No | 2d | Discharged neurologically intact |  |
|  |  |  |  |  |  | Yes | 1h 30m |  | N.A. | Mild left lower limb dysesthesia |  |
| 27 | 1997 | Behringer  AUT | Case report  English | 79M | Digoxin (10mg) | IHCA | 35m | No | N.A. | Demised @ D12 | - Digoxin: 93.6nmol/L |
|  |  |  |  |  |  | Yes | 4h 08m |  | 12d | ARDS, septic shock |  |
| 28 | 1999 | Holzer AUT | Case report  English | 41M | - Verapamil (4.8-6.4g)  - Thiamine nitrate (1g)  - Cyanocobalamin (5mg)  - Timolol (6.25mg)  - Pyridoxine HCl | IHCA | 2h 30m | Yes | 18d | Discharged @ D31 for neuro-psychiatric rehabilitation; return to work @ 6m | Neurologically intact recovery despite long low-flow time (2.5hrs) and secondary transfer   - Verapamil: 630ng/ml |
|  |  |  |  |  |  | No | 4h 50m |  | N.A. | Nil |  |
| 29 | 2000 | Pasic  DEU | Case report  English | 25F | Prajmalium bitartrate (320mg) | IHCA | 45m | No | N.A. | Transferred @ D35 for neuropsychiatry rehabilitation |  |
|  |  |  |  |  |  | Yes | 16h 40m |  | N.A. | - Ataxic gait  - Impaired cognitive function  - Unstable affect, mood disorder |  |
| 30 | 2003 | Durward  CAN | Case report  English | 16F | Diltiazem SR (12g) | IHCA | 51m | No | 13d | Discharged neurologically intact |  |
|  |  |  |  |  |  | No | 2d |  | 15d | - ECLS terminated early for uncontrollable mediastinal haemorrhage, prolonged ileus  - Severe hypotension @ decannulation requiring high-dose epinephrine infusion |  |
| 31 | 2003 | Soltesz USA | Case report  English | 39F | 0.5% bupivacaine | IHCA | 30m | No | 2d | Discharged @ D10 |  |
|  |  |  |  |  |  | Yes | 1h 10m |  | N.A. | Nil |  |
| 32 | 2005 | Rygnestad  NOR | Case report  English | 29F | Verapamil (3.6g) Sotalol (4.8g) | IHCA | 4h | No | 5d | Discharged neurologically intact but with minimal left forearm dysfunction | Neurologically intact recovery despite long low-flow time of 4h of normothermic CPR |
|  |  |  |  |  |  | No | 69h |  | N.A. | - Left forearm compartment syndrome  - Renal failure from rhabdomyolysis - Intestinal bleeding - Transient nerve paralysis |  |
| 33 | 2007 | Kolcz  POL | Case report  English | 15F | Propranolol (550mg)  Verapamil (960mg) | IHCA | 2h | No | 13d | Discharged @ D32 neurologically intact | TPE may augment clearance of cardio-depressive agents and reduce duration of ECLS   - Propranolol: 0.53mcg/ml - Verapamil: 1.06mcg/ml |
|  |  |  |  |  |  | Yes | 70h |  | N.A. | Bleeding during 1st 12 hours - resolved uneventfully with blood and FFP transfusion |  |
| 34 | 2010 | Vivien  FRA | Case report  English | 40F | Flecainide (12g)  Betaxolol (400mg) | IHCA | 1h 30m | No | 3d | Demised @ D3 (brain death) | Heart, liver, kidneys harvested for organ donation – all recipients alive with normal graft function 45 months later   - Flecainide: 7.89umol/L |
|  |  |  |  |  |  | No | 48h |  | 3d | Disseminated intravascular coagulopathy, pulmonary haemorrhage |  |
| 35 | 2011 | Dudley  USA | Case report  English | 37M | Bupivacaine nerve block | IHCA | N.A. | No | N.A. | Demised @ 7h | Cause of death determined to be LA systemic toxicity but autopsy findings and elevated [tryptase] and [bupivacaine] suggest possibility of bupivacaine hypersensitivity instead |
|  |  |  |  |  |  | No | 2h |  | N.A. | N.A. |  |
| 36 | 2011 | Lemoine  USA | Case report  English | 15mo F | Aluminium phosphide fumigation | IHCA | N.A. | No | 2d | Demised @ D2 |  |
|  |  |  |  |  |  | No | 24h |  | 2d | N.A. |  |
| 37 | 2011 | Soumagne  FRA | Case report  English | 46M | Yew (10 leaves) | IHCA | 1h 30m | No | 6d | Transferred with no neurologic/cardiac sequelae |  |
|  |  |  |  |  |  | Yes | 50h |  | 7d | Nil |  |
| 38 | 2014 | Koschny  DEU | Case report  English | 21F | - Carvedilol (1.75g) - Amlodipine (300mg) - Amitriptyline (6g) - Torsemide (500mg) - Ketoprofen (1.5g) - Nicotinic acid (28g) - Gabapentin (16g) | IHCA | 2h | No | 8d | Discharged neurologically intact | Consider integrating ECMO and plasma exchange for unsuccessful resuscitation after severe intoxication |
|  |  |  |  |  |  | No | 92h |  | 8d | Cannula-induced dissection of deep femoral artery leading to compartment syndrome requiring fasciotomy, necrosectomy and wound revision |  |
| 39 | 2014 | Machado  FRA | Case report  English | 55F | White gilled mushrooms | IHCA | 1h 15m | No | 17d | Discharged @ D46 neurologically intact with normal renal function |  |
|  |  |  |  |  |  | Yes | 6d |  | N.A. | Nil |  |
| 40 | 2014 | Meserve  USA | Case report  English | 4M | Cyclophosphamide | IHCA | N.A. | No | N.A. | Demised @ D15 | Hypothesized to be related to cyclophosphamide-induced cardiotoxicity post stem-cell transplant |
|  |  |  |  |  |  | No | 5d |  | 15d | - Hypoxic-ischemic injury - Progressive subdural and intraparenchymal bleeding with mass effect from heparinization - Kidney failure |  |
| 41 | 2014 | Moreno  ESP | Case report  English | 27M | Ajmaline (0.7mg/kg; given as diagnostic challenge for Brugada) | IHCA | 1h 47m | No | 4d | Discharged @ D21 to neuro-rehabilitation centre |  |
|  |  |  |  |  |  | Yes | 43h |  | N.A. | - Altered T5 sensation level  - Paraesthesia and weakness in bilateral lower limbs  - Cognitive dysfunction (slow ideation, minor difficulty with calculation) |  |
| 42 | 2014 | Thooft  BEL | Case report  English | Young F | Yew tree leaves | IHCA | 1h 22m | Yes | N.A. | Discharged neurologically intact | Consider ECPR coupled with therapeutic hypothermia to improve neurologic recovery |
|  |  |  |  |  |  | Yes | 36h |  | 5d | Nil |  |
| 43 | 2014 | Valis  CZE | Case report  English | 39M | Yew needles | IHCA | N.A. | No | N.A. | Discharged bedridden |  |
|  |  |  |  |  |  | Yes | 2d |  | N.A. | Hypoxic encephalopathy with minimal cortical reactivity, generalized myoclonic seizures and serious neurological deficits |  |
| 44 | 2015 | Baum  DEU | Case report  English | 24F | European Yew leaves | IHCA | 3h 45m | Yes | N.A. | Discharged @ D8 to referring hospital |  |
|  |  |  |  |  |  | No | 70h |  | N.A. | Nil |  |
| 45 | 2015 | Enakpene  USA | Case report  English | 25F | Loperamide | N.A. | N.A. | No | N.A. | Demised |  |
|  |  |  |  |  |  | N.A. | N.A. |  | 18h | Nil |  |
| 46 | 2015 | Escajeda  USA | Case report  English | 47M | Metoprolol (10g) | IHCA | 55m | No | 3d | Discharged @ D10 completely recovered | - Metoprolol: 25mcg/ml |
|  |  |  |  |  |  | No | 50h |  | N.A. | Nil |  |
| 47 | 2015 | Mohan  IND | Case series  English | 50 M | Aluminium phosphide (3g) | IHCA | N.A. | No | D8 | Discharged @ D22 well |  |
|  |  |  |  |  |  | Yes | 44h |  | N.A. | Massive bleeding from femoral cannulation site requiring blood transfusion |  |
| 48 | 2015 | Reynolds  USA | Case report  English | 24F | Flecainide (3-4 tablets) | IHCA | 50m | No | 6d | Discharged @ D19 neurologically intact | - Flecainide: 11,085ng/ml |
|  |  |  |  |  |  | No | 5d |  | N.A. | Compartment syndrome requiring fasciotomy with residual impaired mobility and sensation in left leg |  |
| 49 | 2016 | Horn  DEU | Case report  English | 43F | Aspirin (130g) | IHCA | 15m | No | N.A. | Demised | Early calcium supplementation crucial to avoid hypocalcaemia-associated tetany (ionized calcium 0.66mmol/L)   - Aspirin: 84mmol/L |
|  |  |  |  |  |  | No | N.A. |  | N.A. | Refractory hypocalcaemia-induced tetany and arterial spasm caused excessive afterload hampering ECLS implantation |  |
| 50 | 2016 | Kashiwagi  JAP | Case report  Japanese | Teenage M (late teens) | - Cibenzoline (3000mg)  - Sodium valproate (23.6g) - Brotizolam (7mg) - Ibuprofen (3.4g) | IHCA | N.A. | No | 4d | Discharged @ D10 neurologically intact | - Cibenzoline: 6150ng/ml |
|  |  |  |  |  |  | Yes | 3d |  | N.A. | Nil |  |
| 51 | 2017 | Bounes  FRA | Case report  English | 28M | Yew leave capsules | OHCA > IHCA | N.A. | No | N.A. | Transferred @ D8 to Psychiatry |  |
|  |  |  |  |  |  | Yes | 48h |  | N.A. | Nil |  |
| 52 | 2017 | Farag  DEU | Case report  English | 20F | Yew | IHCA | 1h 30m | No | N.A. | Discharged neurologically intact |  |
|  |  |  |  |  |  | No | 5d |  | N.A. | - Persistent renal failure requiring dialysis  - Paralytic ileus requiring ileostomy  - Pancreatic necrosis requiring distal pancreatectomy and splenectomy |  |
| 53 | 2017 | Hong  KOR | Case series  English | 47M | Aconite (20 tablets) | IHCA | 10m | No | N.A. | Discharged @ D10 |  |
|  |  |  |  |  |  | Yes | 33h |  | N.A. | Nil |  |
| 54 | 2017 | Hughes  USA | Case report + review article  English | 14F | Diphenhydramine | IHCA | N.A. | No | 3d | Discharged with mild ataxia, slight cognitive dysfunction |  |
|  |  |  |  |  |  | Yes | 4d |  | N.A. | Nil |  |
| 55 | 2017 | Ito  JAP | Case series + review article  English | 20F | - Risperidone (122mg)  - Flunitrazepam (50mg) - Aspirin (52.8g) | IHCA | N.A. | No | 12d | Transferred | - [risperidone]: 9.6ng/ml - [9OH-RIS]: 127.6ng/ml |
|  |  |  |  |  |  | No | 2d |  | N.A. | Nil |  |
| 56 | 2017 | Kacirova  CZE | Case report  English | 14F | Metoprolol (1g)  Propafenone (1.5-3g) | IHCA | N.A. | Yes | 6d | Demised (brain death) | Transfer to closest ECMO centre took 33 mins.   - Metoprolol: 2630ng/ml - Propafenone: 2500ng/ml |
|  |  |  |  |  |  | Yes | 6d |  | 6d | - Acute right lower limb ischemia from interruption of blood flow by cannula requiring surgical revision |  |
| 57 | 2017 | Napp  DEU | Case report  English | 24M | Venlafaxine (9g) | IHCA | N.A. | Yes | N.A. | Discharged neurologically intact | ECMO triple-cannulation VA-PA (pulmonary arterial) set-up + Impella micro-axial pump in LV maintained antegrade transpulmonary flow, bypassing biventricular heart and lung failure |
|  |  |  |  |  |  | No | 6d |  | 28d | Nil |  |
| 58 | 2017 | Nishimura JAP | Case report  English | 21M | Amitriptyline | IHCA | 30m | No | N.A. | Transferred @ D54 to rehabilitation hospital with serious neurological disability | - Amitriptyline: 306.2ng/ml |
|  |  |  |  |  |  | Yes | N.A. |  | N.A. | - Uncontrolled bleeding from femoral catheter site - Comatose (E2V2M4) but bilateral frontal lobe and insula damage not compatible with hypoxic damage  - Intestinal emphysema causing abdominal compartment syndrome - Small bowel obstruction/strangulation requiring laparoscopic resection |  |
| 59 | 2017 | Sood  USA | Case report  English | 9M | Brown recluse spider | IHCA | 1h 30m | No | 8d | Discharged neurologically intact |  |
|  |  |  |  |  |  | Yes | 48h |  | N.A. | Nil |  |
| 60 | 2018 | Labarinas USA | Case report  English | 17M | Diphenhydramine (20g) | IHCA | 9m | No | 9d | Discharged @ D17 with good functional outcomes | Endoscopic gastrointestinal decontamination performed to remove suspected bezoar |
|  |  |  |  |  |  | Yes | 5d |  | 9d | Hypoxic ischemic injury in posterior globus pallidus, putamen and ventrolateral thalami but had full recovery with rehabilitation @ D17 |  |
| 61 | 2018 | Lehoux  USA | Case report  English | 3F | Aluminium phosphide pellets | IHCA | 1h | No | 19d | Complete recovery | Importance of multidisciplinary teamwork in the care of acutely ill child |
|  |  |  |  |  |  | No | 15d |  | 30d | Pulmonary oedema due to LV distension - resolved by balloon atrial septostomy |  |
| 62 | 2018 | Marano  ITA | Case report  English | 16F | Propafenone (1.8g) | IHCA | 1h | Yes (ECPR in progress*) | N.A. | Discharged neurologically intact | *Early "on-site" ECPR performed in peripheral hospital ER allowed patient to be stabilized during transport to closest ECMO centre; contributed to patient survival |
|  |  |  |  |  |  | No | 4d |  | 6d | Nil |  |
| 63 | 2019 | Hantson  BEL | Case report  English | 18F | Extended-release Flecainide (18g) | IHCA | 35m | No | 9d | Discharged with normal ECG, echocardiography | "After the development of a refractory shock, ACLS was commenced ... and electric shocks for VT and VF ... there was no change in hemo-dynamic conditions and ECLS was initiated ...”   - Flecainide: 2386ug/L |
|  |  |  |  |  |  | Yes | 6d* |  | N.A. | - Acute limb ischemia  - *Hemodynamic instability @ 48h post-ECLS withdrawal necessitating reintroduction of ECLS (eventually withdrawn on D8) |  |
| 64 | 2019 | Kato  JAP | Case report  English | 43F | - Caffeine (15,360mg)  - Ibuprofen (28.8g)  - Amitriptyline (2.5g)  - Risperidone (48mg) - Lamotrigine (400mg) | IHCA | 26m | No | N.A. | Discharged @ D45 completely recovered | - Caffeine: 251mcg/ml - Ibuprofen: 25.9mcg/ml - Amitriptyline: 0.2mcg/ml |
|  |  |  |  |  |  | Yes | 3d |  | N.A. | Transient right peroneal nerve paralysis from prolonged limb compression |  |
| 65 | 2019 | Nagasawa  JAP | Case report  English | 49F | - Boric acid  - Mirtazepine (1950mg)  - Sennosides (780mg) | IHCA | N.A. | No | 30d | ADL-dependent @ 6m: cannot walk but able to swallow food, speak single words | ECPR, even in prolonged cardiac arrest induced by overdose, is medically, ethically and economically challenging   - Boric acid: 560.49mg/L - Mirtazepine: 1270ng/ml |
|  |  |  |  |  |  | No | 6d |  | 39d | - Leg necrosis post-cannulation requiring amputation - Hypoperfusion cerebral ischemia of bilateral white matter and caudate nuclei - Bilateral corneal ulcers from boric acid poisoning |  |
| 66 | 2019 | Stros  CZE | Case report  Czech | 25M | Yew needles (50g) Escitalopram (250mg) | IHCA | 49m | No | N.A. | Demised (brain death) |  |
|  |  |  |  |  |  | Yes | 4d |  | 6d | - Right lower limb ischemia due to arterial rupture at cannulation site + posterior tibial artery thrombosis requiring angiographic recannalisation |  |
| 67 | 2019 | Voizeux  FRA | Case report  English | 22M | MDMA, cannabis | IHCA | N.A. | No | 20d | Survival | “Because of sustained hemodynamic instability without rhythmic recovery (refractory cardiac arrest), ECLS was implanted. We were able to rapidly regulate our patient's temperature and we weaned all hemodynamic support in the first week of hospitalisation.” |
|  |  |  |  |  |  | Yes | 4d |  | N.A. | - ARDS from ventilator-associated pneumonia  - rhabdomyolysis  - opioid-specific withdrawal  - multi-organ failure |  |
| 68 | 2020 | Hermes  SUI | Case series + review article  English | 30F | Yew | IHCA | 1h | No | N.A. | Complete recovery |  |
|  |  |  |  |  |  | Yes | 71h |  | 11d | Nil |  |
| 69 | 2020 | Sato  JAP | Case report  English | 53F | Cibenzoline (6.9g) | IHCA | 20m | No | 5d | Discharged @ D9 to Psychiatry | - Cibenzoline: 6000ng/ml |
|  |  |  |  |  |  | Yes | 3d |  | N.A. | Nil |  |
| 70 | 2021 | Devanand  AUS | Case report  English | 24F | Propranolol (2.4g) Diazepam (15mg) | IHCA | 1h 07m | No | N.A. | Discharged neurologically intact |  |
|  |  |  |  |  |  | No | 2d |  | 9d | Nil |  |
| 71 | 2021 | Ferry  SUI | Case report  English | 14F | Chloroquine (3g) | OHCA | 1h 06m | No | 7d | Transferred @ D11 to local rehabilitation hospital | - Chloroquine: 0.06umol/L |
|  |  |  |  |  |  | Yes | 46h |  | 11d | Right leg compartment syndrome from right common femoral artery stenosis at cannula insertion site requiring fasciotomy |  |
| 72 | 2021 | Giuliano USA | Case report  English | 16F | Amlodipine | IHCA | 1h 15m | No | 5d | Transferred @ D27 to Psychiatry with intact neurological and renal function |  |
|  |  |  |  |  |  | No | 4d |  |  | - Microhaemorrhages in supra/infratentorial brain  - Critical illness peripheral neuropathy |  |
| 73 | 2021 | Itoh  JAP | Case report  English | 30+F | Cibenzoline | OHCA > IHCA | 38m | No | N.A. | Discharged neurologically intact | Abdominal surgery (for gastric perforation from mechanical CPR) done even while ECLS ongoing   - Cibenzoline: 3868ng/ml |
|  |  |  |  |  |  | No | N.A. |  | N.A. | Gastric perforation from mechanical CPR |  |
| 74 | 2021 | Li  CHN | Case report  English | 56M | White mushrooms (500g) | IHCA | N.A. | No | N.A. | Discharged @ D43 with complete recovery |  |
|  |  |  |  |  |  | Yes | 6d |  | 25d | Nil |  |
| 75 | 2021 | Shen  CHN | Case report  English | 15F | Aluminium phosphide | IHCA | N.A. | No | 83h | Recovered without HIE or end-organ damage |  |
|  |  |  |  |  |  | Yes | 65h |  | N.A. | Osteo-facial compartment syndrome of lower limb requiring debridement |  |
| 76 | 2021 | Vlok  ZAF | Case series  English | Middle-aged F | Caffeine | IHCA | N.A. | No | N.A. | Discharged @ D10 neurologically intact | Benefit of ECLS in low-to-middle income country is debatable though effective |
|  |  |  |  |  |  | Yes | 4d |  | N.A. | N.A. |  |
| 77 | 2021 | Yasuda  JAP | Case report  English | 43 M | Caffeine (20g) | IHCA | 3m | No | 4d | Discharged @ D21 to Psychiatric facility | Advance placement of catheter sheaths can enable rapid introduction of ECMO |
|  |  |  |  |  |  | Yes | 2d |  | N.A. | N.A. |  |
| 78 | 2021 | Yopes  USA | Case report (poster)  English | 52F | Ethylene glycol | IHCA | 45m | No | N.A. | Discharged with no cardiac/ neurologic sequelae but remained dialysis-dependent @ 2months |  |
|  |  |  |  |  |  | Yes | 9d |  | N.A. | End-stage renal failure |  |
| 79 | 2021 | Zickler  DEU | Case series  English | 60M | Amitriptyline (4g) | OHCA | 1h 25m | No | 7d | Discharged @ D23 with intact neurological, renal and liver function | Dramatic decline in drug levels suggest potentially lifesaving role of CytoSorb for acute intoxication causing multiple organ failure. Easy to use Cytosorb in combination with VA-ECMO + CRRT. |
|  |  |  |  |  |  | No | 5d |  | N.A. | Nil |  |
| 80 | 2022 | Chen  CHN | Case report  English | 60M | *Macleaya cordata*  (Chinese herbal medicine) | IHCA | N.A. | No | N.A. | Discharged @ D12 neurologically intact |  |
|  |  |  |  |  |  | Yes | 4d |  | N.A. | N.A. |  |
| 81 | 2022 | Humphreys  AUS | Case report  English | 25F | Nortriptyline (2500mg) | IHCA | 1h 30m | Yes | N.A. | Discharged @ D29; return to work within 3m | - Nortriptyline @ 16H post-ingestion: 890mcg/L |
|  |  |  |  |  |  | Yes | 3d |  | N.A. | N.A. |  |
