# Supplementary material for: Extracorporeal cardio-pulmonary resuscitation in poisoning: A scoping review article
Source: Resusc Plus. 2023 Feb 18;13:100367. doi: 10.1016/j.resplu.2023.100367 (PMC9969255; doi:10.1016/j.resplu.2023.100367)
Supplement: Supplementary data 2 [file mmc2.docx]

Supplementary 2: Cases with Ambiguity

| # | Year | Author  Country | Study type  Language | Age, sex | Toxins (dose)   - peak levels | IHCA/ OHCA | Time from arrest to ECPR | Transfer to ECLS facility required | Intubation duration | Outcomes | Remarks |
| --- | --- | --- | --- | --- | --- | --- | --- | --- | --- | --- | --- |
|  |  |  |  |  |  | VT/VF* | ECLS duration |  | ICU LOS | Complications |  |
| 82 | 1982 | Mattox  USA | Case series  English | N.A. | Intravenous injection of multiple illicit drugs | OHCA | N.A. | No | N.A. | Demised | "… patient fully awake upon arrival, although cardiac output was being maintained by external cardiac massage by emergency medical technicians." |
|  |  |  |  |  |  | No | N.A. |  | N.A. | N.A. |  |
| 83 | 1993 | Goodwin  USA | Case report  English | 18m F | Desipramine hydrogen chloride | OHCA > IHCA | 4h | No | 4d | Discharged neurologically intact except for mild generalized weakness | "Due to the persistent bradycardia and unresponsive hypotension, the ECMO transport team was consulted … CPR and hand ventilation were continued.” |
|  |  |  |  |  |  | Yes | 48h |  | N.A. | Mild generalized weakness at discharge that resolved on follow up evaluation |  |
| 84 | 2007 | Fujita  JAP | Case series  English | 53M | Aconite roots | IHCA | N.A. | No | N.A. | Discharged | “case 1 requiring additional PCPS because of unstable hemodynamics” |
|  |  |  |  |  |  | Yes | N.A. |  | N.A. |  |  |
| 85 | 2008 | Haas  DEU | Case report  English | 1d  neonate | Amiodarone infusion (given 41.25mg) | IHCA | N.A. | No | N.A. | Discharged @ D9; neurologically intact recovery @ 1 year | “continuous deterioration of the clinical status requiring repetitive cardiothoracic massage and additional bolus administrations of epinephrine to maintain adequate blood pressure and heart rate.” |
|  |  |  |  |  |  | Yes | 36 h |  | N.A. | N.A. |  |
| 86 | 2008 | Terui  JAP | Case report  English | 41M | Aconite leaves | N.A. | N.A. | No | N.A. | Survival | “Patients 1 occurred VT and VF… resistant to treatment with lidocaine and electrical cardioversion. These patients required PCPS due to unstable hemodynamics. Patient 1 required additional CPB.” |
|  |  |  |  |  |  | Yes | N.A. |  | N.A. |  |  |
| 87 | 2011 | De Rita  ITA | Case report  English | 33d F | Verapamil (0.1mg/kg) Propranolol (2.5mg)  - Meds administered for SVT | IHCA | 5h | No | 17d | Discharged neurologically intact | "Refractory cardiocirculatory collapse persisted and, approximately 5h after no clinical improvement, the baby was transferred to the Pediatric Cardiac Surgery Unit during closed chest cardiac massage for ECMO support" |
|  |  |  |  |  |  | Yes | 100h |  | 17d | Nil |  |
| 88 | 2011 | Elkalioube  FRA | Case report  English | 22F | Tramadol (4.5g) | N.A. | N.A. | No | N.A. | Discharged @ D35 neurologically intact | "Repeated episodes of cardiac arrest required CPR and immediate ICU transfer where refractory circulatory shock was diagnosed, requiring ECLS by VA-ECMO" |
|  |  |  |  |  |  | No | 7d |  | N.A. | N.A. |  |
| 89 | 2017 | Vo  USA | ECG vignette  English | 50+F | Aconite in home-brewed Chinese herbal tea | IHCA | 3h | No | N.A. | N.A. | "The patient’s condition rapidly deteriorated and she developed … intermittent pulselessness requiring CPR. … After 3 hours of attempted resuscitation, VA-ECMO was instituted." |
|  |  |  |  |  |  | Yes | N.A. |  | N.A. | Multiorgan failure from prolonged resuscitation |  |
| 90 | 2020 | Chien  TWN | Case report  English | 36F | Hymenoptera bee-sting | IHCA | 25m | No | 96d | Discharged @ D101 clinically stable | Hypersensitivity myocarditis from bee sting is rare. Consider orthotopic heart transplantation if patient does not recover and cannot be weaned from mechanical circulatory support. |
|  |  |  |  |  |  | Yes | 58d |  | N.A. | - Mediastinal bleeding - Sternal wound infection with abscess - pressure sore infections - Hyperbilirubinemia |  |
| 91 | 2020 | Ikejiri  JAP | Case report + review article  English | 19M | Amitriptyline   - 2902ng/ml | IHCA | 40m | No | 5d | Discharged neurologically intact | "… he had another cardiopulmonary arrest shortly afterward. VA-ECMO was initiated … based on the prediction that refractory arrhythmia and cardiac arrest could easily recur until the TCA-associated cardiotoxicity had disappeared" |
|  |  |  |  |  |  | Yes | 27h |  | 15d | Nil |  |
| 92 | 2021 | Forsberg  SWE | Case report  English | 48M | Venlafaxine (20g)   - 12.6mg/L   Zolpidem (450mg) Propiomazine (250mg) | IHCA | 2h | Yes | 8d | Discharged neurologically intact | “The patient’s circulatory status and blood pressure were, however, inadequate … The patient was then transported by helicopter to a tertiary hospital using mechanical CPR and ongoing epinephrine infusion.” |
|  |  |  |  |  |  | No | 32h |  | 30d | Bleeding from femoral artery catheter requiring blood transfusions |  |
| 93 | 2022 | Schreiber  AUT | Case report  English | 39M | Yew leaves and branches (30g) | OHCA > IHCA | N.A. | No | 7d | Discharged @ D23 neurologically intact | “… we immediately performed ECLS via VA-ECMO, which was implanted during ongoing resuscitation with an automated chest compression system.” |
|  |  |  |  |  |  | Yes | 39h |  | 12d | N.A. |  |
